# Localization of Organelle Proteins Using Data-Independent Acquisition (DIA-LOP)

**DOI:** 10.1016/j.mcpro.2025.101047

**Published:** 2025-08-07

**Authors:** Kieran McCaskie, Charlotte Hutchings, Renata Feret, Yong-In Kim, Lisa Breckels, Michael Deery, Kathryn Lilley

**Affiliations:** Department of Biochemistry, Cambridge Centre for Proteomics, University of Cambridge, Cambridge, UK

**Keywords:** subcellular spatial proteomics, mass spectrometry, data-independent acquisition, machine learning, localisation of organelle proteins

## Abstract

Subcellular localization within the proteome fundamentally influences cellular processes; however, the development of high-throughput techniques to allow proteome-wide mapping of the cell has proven difficult. Here we present DIA-LOP, an approach capable of high-throughput spatial proteome mapping with in-depth subcellular resolution. This unified framework integrates differential-ultracentrifugation (DC) with ion-mobility-based data-independent acquisition mass spectrometry, alongside data processing using DIA-NN and spatial analysis within the pRoloc bioinformatics pipeline. We obtain the largest DIA-based subcellular proteomics map, with 8242 protein identifications across 13 organellar compartments in U-2 OS cells. Within the same experimental pipeline, we compare DC fractionation with an alternate detergent-based protocol using either DIA or data-dependent acquisition (DDA) mass spectrometry approaches, highlighting the increased subcellular resolution of the DC approach and the increased proteome coverage when DIA is applied. We demonstrate the ability of DIA-LOP to inform clinical studies by identifying and mapping disease-related proteins within our osteosarcoma cell model. With impressive coverage and resolution, DIA-LOP provides a straightforward, high-throughput tool for biochemical discovery. This study thus informs potential users of subcellular proteomics strategies that employ biochemical fractionation of the optimal workflows to achieve high proteome coverage and subcellular resolution.

## Highlights

• DIA-LOP is a high-throughput method for in-depth subcellular proteome mapping.
• This approach demonstrates high performance when compared to previous subcellular spatial proteomics workflows in terms of coverage and resolution.
• DIA-LOP can be utilized to provide insight into protein complexes and disease-related proteins.

## Graphical Abstract

**Figure:**
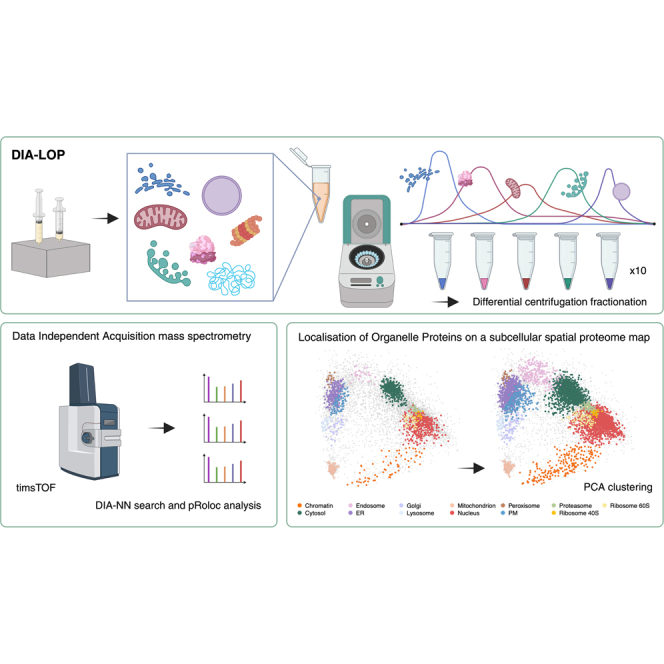

## In Brief

Here, we present DIA-LOP, an approach capable of in-depth subcellular spatial proteome mapping. This approach integrates differential-ultracentrifugation (DC) fractionation with ion-mobility-based data-independent acquisition (DIA) mass spectrometry, alongside data search within DIA-NN, and robust spatial analysis with the pRoloc bioinformatics pipeline. DIA-LOP excels when compared to other workflows regarding coverage and resolution, and provides a straightforward, high-throughput tool for biochemical discovery.

Compartmentalization of cells into organelles allows separate biochemical processes to occur simultaneously, as the subcellular localization of a protein influences its function by defining the surrounding physical conditions and biomolecules available for interaction. Moonlighting proteins can utilize this effect by fulfilling different roles depending on their localization in the cell, and mislocalization is linked with disease (1, 2). As part of routine cellular activity, proteins can re-locate between organelles (3, 4, 5), which alters the local availability of the re-locating protein within its respective organelle; a phenomenon that may occur without any change in its total cellular abundance. Although it is now possible to predict localization to some subcellular compartments using computational tools (6), these methods cannot account for the well-established context-dependent and dynamic nature of protein localization (7, 8, 9). Therefore, high-throughput protocols that can map the subcellular localization of thousands of proteins in a single experiment provide a powerful way to enhance our understanding of protein function and dynamic cellular processes. The optimization of techniques for profiling the subcellular spatial proteome is imperative, to resolve proteomic changes where overall abundance stays constant but localization changes.

The creation of cell-wide subcellular proteome maps allows for unbiased exploration in comparison to targeted methods and is useful for multiple experimental applications, including data-driven hypothesis generation, validation of steady-state localization (including for multi-localized proteins), and comparison of different biological conditions to elucidate protein dynamics. Proximity labelling and organelle immunoprecipitation have been used to create cellular maps (10, 11); however, these techniques require genetic manipulation, which limits their use in some organisms and may lead to artifactual protein behavior (Table 1). Furthermore, to be truly proteome-wide, many individual experiments need to be performed with a different tagged protein per experiment. This biases localization data toward the sampled compartments and severely limits the throughput. These lower-throughput techniques lack the agility for large-scale practical use, especially for the comparison of multiple conditions within protein dynamics and biomedical studies.

**Table 1:** Comparison of subcellular proteome methods

Method	Number of proteins	Cell line	Number of experiments	Genetic manipulation
DIA-LOP	8242	U-2 OS	1 triplicate set of differential centrifugation	No
Global organelle profiling (Hein *et al.,* 2025) (11)	8538	HEK293T	58 triplicate sets of immunoprecipitation	Yes, CAS9 required to tag organelles for pull down
BioID (Go *et al.,* 2021) (10)	4145	HEK293	192 duplicate sets of proximity labelling	Yes, constructs required for biotin ligase fusion proteins

Prominent high-throughput methodologies are often based upon De Duve’s principle, which states that proteins from the same compartment behave similarly when subject to biochemical fractionation, allowing proteins to be mapped by comparison of their profiles across fractions of cell lysate (12). Such techniques include, localization of organelle proteins using isotope tagging (LOPIT) (13, 14, 15, 16, 17), protein correlation profiling (PCP) (18), dynamic organellar maps (DOMs) (19, 20, 21), sequential detergent-based solubilization (22), SubCellBarCode (23), COLA (24), and Prolocate (25).

Significant advances in technical capability have occurred across the LOP (Localization of Organelle Proteins) series of protocols, including LOPIT (13), hyperLOPIT (15), dLOPIT (17) and LOPIT-DC (16), which provide the subcellular localization of thousands of proteins simultaneously. These techniques utilize precise cellular lysis and reproducible centrifugation-based fractionation techniques to yield biochemical fractions, alongside streamlined data analysis within the pRoloc bioinformatics pipeline (26). The user can design an experiment comprised of techniques from these modular workflows to suit their technical constraints and biological requirements. Although these techniques achieve impressive resolution, isotope tagging using TMT labels necessitates a data-dependent acquisition (DDA) workflow. As a result, these methods have limited protein coverage due to the random peak selection of DDA, as well as isobaric tagging-related issues, including the distortion of quantitative data due to precursor co-isolation (27, 28) and instances of missing values between different TMT plexes. Additionally, the pooling of multiplexed TMT samples is followed by an offline pre-fractionation step to ensure in-depth proteome coverage, which extends the time required for the protocol and results in a high number of samples for mass spectrometry analysis. TMT labels necessitate a high amount of input protein material for the labeling protocol, meaning that a high number of cells need to be collected, which limits the use of isotope-labeled methods on samples such as primary cells or patient samples.

Here we present DIA-LOP, an advanced streamlined approach within the LOP framework, which integrates differential-ultracentrifugation (DC) fractionation with ion-mobility-based data-independent acquisition (DIA) mass spectrometry, alongside data search within DIA-NN (29), and robust spatial analysis with the pRoloc bioinformatics pipeline. Within this pipeline we present a mixed imputation strategy to deal with the biologically expected missing values across our separation gradient. This provides the key to interpreting DIA-MS data generated using a high number of biochemical fractions, to unlock considerable resolution and depth. DIA-LOP builds upon the advantages of the previous LOP protocols while utilizing DIA, which features wide m/z windows to capture an increased amount of fragment information compared to previous DDA protocols. Throughput is increased in various ways compared to DDA protocols: here we no longer require isotope labels or the associated offline-prefractionation steps and can utilize a shorter mass spectrometry gradient, to streamline the sample preparation and acquisition phases. Cost efficiency of the sample preparation is improved as expensive tagging reagents are not required, and lower amounts of cellular input material can be used, as the amount of protein required for TMT labeling is no longer a bottleneck. DIA-LOP data analysis involves a library search using the freely available DIA-NN software before subsequent, subcellular spatial analysis in R using pRoloc, to allow maximum flexibility and traceability. The LOP fractionation protocol is based upon the previous LOPIT-DC method, which separates the cell into 10 fractions. This is a higher figure compared to other DIA mapping methods (21, 22), and gives more granular profiles allowing for higher resolution maps. All prominent techniques focus on mapping a single cell type, which is optimal, since the map can then be interpreted without other cell types to confound conclusions. Despite this, a recent tissue-wide DIA map has been created using correlation profiling coupled with crude pestle, mortar, and blender-based lysis (30); this study is difficult to compare with other techniques due to the heterogeneous cell types collected, and the limited coverage due to the use of dated MS equipment. In contrast, here we use precise ball-bearing based cell lysis and avoid the use of detergents in the fractionation buffer to reliably keep the organelles intact. Furthermore, by using a 62.5 min liquid chromatography gradient with a μPAC column coupled with ion mobility separation, sensitivity is ensured whilst retaining a high throughput.

We obtain and compare cellular maps generated with DIA-LOP and LOPIT-DC on the exact same human osteosarcoma U-2 OS cells. We evaluate the impact of employing a DIA workflow on global experiment-wide depth and resolution and further demonstrate the advantage of the DC and DIA combination through comparison to detergent-based fractionation within the same experimental pipeline. Finally, we highlight the capability of DIA-LOP to achieve suborganellar resolution and localize sarcoma-associated proteins identified within the osteosarcoma cell model, to demonstrate potential clinical applications. This expands the capabilities of the modular LOP suite, with streamlined label-free sample preparation allowing for reduced input material, high-throughput DIA-MS, and novel computational analysis strategies integrated within the familiar pRoloc package workflow.

## Experimental Procedures

### Experimental Design and Statistical Rationale

To generate all subcellular spatial proteomic maps, we used three biological replicates from different cell culture flasks. The use of three replicates ensured confidence in protein identifications, as only proteins found in all replicates were retained in the spatial analysis. For each replicate, we generated fractions from the cell lysate, which were aliquoted into two parts for both TMT labelled DDA and label-free DIA analysis on the same samples. For DIA-LOP, we ran aliquots of each fraction of each replicate in a separate MS run (3 × 10 MS runs). For LOPIT-DC, we TMT labelled each lysate fraction and then pooled the 10 fractions before Ultra-Performance Liquid Chromatography (UPLC) separation and orthogonal combination to give 18 samples to be analyzed by MS per replicate (3 × 18 MS runs). Standard FDR thresholds were upheld during database searches, with only high-confidence PSMs with an FDR lower than 1% retained for analysis (31). DDA experiments utilized TMT labels, and quality control involved filtering based on average reporter ion signal-to-noise, co-isolation interference, and percentage synchronous precursor selection (SPS) mass matches, whilst DIA quality control involved removal of ambiguous protein groups, intensity high-pass filtration, and global missing value filtration concerning missingness within a fraction and across the separation profile. Principal component analysis was used to assess the variance across the datasets, with QSep and F1 scores calculated to quantify spatial map resolution. QSep analysis relies on the comparison of the average Euclidean distance of the full, n-dimensional protein profiles within and between subcellular marker clusters to quantify the distance between each cluster alongside the tightness of clustering (32). F1 scores were computed as the harmonic mean of the precision (a measure of exactness) and recall (a measure of completeness), scaled from 0 to 1; a higher F1 score indicates that the markers are consistently assigned to the correct subcellular localization by the classifier (33).

### Cell Culture

U-2 OS cells were grown at 37 °C in McCoy’s 5A modified medium (ThermoFisher) supplemented with 10% foetal bovine serum (Merck). Per LOP replicate (material for DIA-LOP and LOPIT-DC), one 245-mm^2^ dish was used. Per detergent replicate (material for DIA-detergent and DDA-detergent), one 150-mm^2^ diameter dish was used. Upon confluence, cells were harvested by trypsinization at 37 °C for 7 min and washed three times by resuspending cells in Dulbecco's Phosphate-Buffered Saline (Merck) and centrifuging at 300 RCF for 5 min at 4 °C.

### Differential Ultracentrifugation (DC) Fractionation

DC fractionation was performed as in Geladaki *et al.* 2019 (16). Harvested cells were resuspended in 1 ml of lysis buffer (250 mM sucrose, 10 mM HEPES pH7.4, 2 mM EDTA, 2 mM magnesium acetate, and cOmplete protease inhibitors sourced from Sigma Aldrich; 11836170001 and 11873580001), before lysis in a ball bearing homogenizer (Isobiotec). Cells were passed through a clearance of 12 μm in the ball bearing chamber 25 times while on ice.

The lysate was separated into 11 fractions by differential ultracentrifugation using an Eppendorf 5425R and Beckman Optima MAX-XP Ultra as outlined in Supplemental Table S1. After each sequential spin, the supernatant was transferred to a new tube for centrifugation. The final supernatant was precipitated with 6 volumes of acetone at −20 °C overnight. The obtained precipitated pellet and membrane pellets were resolubilised in 8 M urea, 0.2% SDS, and 50 mM HEPES pH 8.5.

### Sequential Detergent Fractionation

Detergent fractionation was adapted from the method presented in Martinez-Val *et al.*, 2021 (22). Harvested cells were resuspended in 540 μl of buffer A (350 mM sucrose, 30 mM HEPES pH7.4, 1 mM EDTA, 2 mM MgCl_2_, 15 mM NaCl and cOmplete protease inhibitors sourced from Sigma Aldrich; 11836170001 and 11873580001).

Fraction 1: 60 μl of 0.15% digitonin solution was added before rotation for 30 min at 4 °C. The sample was centrifuged at 500 RCF for 3 min in an Eppendorf 5425R centrifuge, and the supernatant was recovered and transferred to a clean tube. The pellet was washed twice using 1 ml of buffer A.

Fraction 2: The pellet was resuspended in 540 μl of buffer A, and 60 μl of 1.4 M NaCl was added before rotation for 15 min at 4 °C. The centrifugation and wash steps were repeated as for fraction 1.

Fraction 3: The pellet was resuspended in 570 μl of buffer A and 30 μl of 10% Tween-20 was added before rotation for 15 min at 4 °C. The centrifugation and wash steps were repeated as for fraction 1.

Fraction 4: The pellet was resuspended in 540 μl of buffer B (20% glycerol, 30 mM HEPES pH7.4, 1 mM EDTA, 2 mM MgCl_2_, 15 mM NaCl, cOmplete protease inhibitor tablets) and 60 μl of 10% dodecyl maltoside was added, before rotation for 15 min at 4 °C. The centrifugation and wash steps were repeated as for fraction 1.

Fraction 5: The pellet was resuspended in 540 μl of buffer C (30 mM HEPES pH7.4, 1 mM EDTA, 2 mM MgCl_2_, 15 mM NaCl, cOmplete protease inhibitor tablets) and 60 μl of 5 M NaCl and 1 μl of Benzonase nuclease was added, before rotation for 15 min at 4 °C. The centrifugation and wash steps were repeated as for fraction 1.

Fraction 6: The pellet was resuspended in 522 μl of buffer C, and 60 μl of 1.4 M NaCl and 18 μl of 10% SDS were added, before rotation for 15 min at 4 °C. The centrifugation and wash steps were repeated as for fraction 1.

All 6 fractions were centrifuged for 5 min at 20,000 RCF, and the supernatants were extracted for analysis.

### DDA Sample Preparation, Mass Spectrometry, and Database Search

25 μg of protein from each fraction was reduced by shaking for 1 h with 10 mM of DTT (dithiothreitol) and alkylated by shaking for 2 h in the dark with 25 mM of IAA (iodoacetamide). Samples were precipitated with 6 volumes of acetone at −20 °C overnight.

Precipitated proteins were recovered by centrifugation at 15,000 RCF for 20 min at 10 °C, before removal of the supernatant and resuspension in 25 μl of 100 mM TEAB (Triethylamonium bicarbonate) to give a concentration of 1 g/l. Each protein fraction was digested with trypsin overnight at 37 °C, with a trypsin to protein ratio of 1:20 (w/w). 10-plex tandem mass tags (TMT Thermo) were used to label the LOPIT-DC fractions, whilst 6-plex TMT labels (Thermo) were used for the detergent fractions. 42 μl of anhydrous acetonitrile was added to each 0.8 mg TMT vial. 10.5 μl of dissolved TMT label reagent was added to its corresponding 25 μl peptide sample and incubated for 1 h at room temperature. 3 μl of 5% hydroxylamine was used to quench the reaction for 20 min. The fractions were pooled into a single tube and lyophilized. Desalting was performed using Pierce Peptide Desalting Spin Columns (Thermo) according to the manufacturer’s instructions.

The following LC conditions were used for the offline pre-fractionation of the TMT samples: desalted peptides were resuspended in 0.1 ml 20 mM ammonium formate (pH10) + 4% (v/v) acetonitrile. Peptides were separated using an Acquity Premier UPLC system (Waters). Peptides were loaded onto an Acquity UPLC BEH C18 column (Waters; 2.1 mm i.d. x 150 mm, 1.7 μm particle size), and profiled with a linear gradient of 5 to 100% B over 75 min, at a flow rate of 0.244 ml/min. Buffer A was 20 mM ammonium formate (pH10) in H_2_O and buffer B was 20 mM ammonium formate in 80% acetonitrile and 20% H_2_O (pH10). Chromatographic performance was monitored by sampling the eluate with a PDA detector at wavelengths of 210 and 280 nm. Peptides were collected in 1 min increments. 36 fractions were collected from the moment that the peptides began to elute. For analysis, the fractions were orthogonally combined into 18 samples, lyophilised, and resuspended in 0.1% formic acid for mass spectrometry (MS) analysis. 1 μg of each combined fraction was injected by the HPLC autosampler and analyzed by the method described below.

Experiments were performed using a Dionex Ultimate 3000 RSLC nanoUPLC (Thermo Fisher Scientific Inc) system and a Fusion Lumos Orbitrap mass spectrometer (Thermo Fisher Scientific). Peptides were loaded onto a pre-column (Thermo Scientific PepMap 100 C18, 5 mm particle size, 100 Å pore size, 300 mm i.d. × 5 mm length) from the Ultimate 3000 autosampler with 0.1% formic acid for 3 min at a flow rate of 15 μl/min. After this period, the column valve was switched to allow elution of peptides from the pre-column onto the analytical column. Separation of peptides was performed by C18 reverse-phase chromatography at a flow rate of 300 nl/min using a Thermo Scientific reverse-phase nano Easy-spray column (Thermo Scientific PepMap C18, 2 mm particle size, 100A pore size, 75 μm i.d. × 50 cm length). Solvent A was H_2_O + 0.1% formic acid, and solvent B was 80% acetonitrile, 20% H_2_O + 0.1% formic acid. The linear gradient employed was 2 to 40% B in 103 min (total LC run time was 120 min, including a high organic wash step and column re-equilibration).

Peptides eluted from the LC were sprayed into the mass spectrometer using an Easy-Spray source (Thermo Fisher Scientific Inc). Briefly, all mass-to-charge (m/z) values of eluting peptide ions were measured in the Orbitrap mass analyzer, set at a resolution of 120,000, and were scanned between 380 and 1500 m/z. Data-dependent MS/MS scans (Top Speed) were employed to automatically isolate and fragment precursor ions by collision-induced dissociation (CID, Normalized Collision Energy (NCE): 35%), which were analyzed in the linear ion trap. Singly charged ions and ions with charge states greater than 7 or unassigned charge states were excluded from being selected for MS/MS, and a dynamic exclusion window of 70 s was employed. The 10 most abundant fragment ions from each MS/MS event were selected for a further stage of fragmentation by Synchronous Precursor Selection-MS3 (SPS-MS3) in the high-energy collision cell using HCD (High energy Collisional Dissociation, NCE: 65%). The m/z values and relative abundances of each reporter ion (mass range from 100-500 Da) in each MS3 step were measured in the Orbitrap analyzer, which was set at a resolution of 50,000.

For DDA analyses, raw files were processed with Proteome Discoverer 3.0 (Thermo Fisher Scientific), to perform peptide sequence searches against the human SwissProt database with 20,399 entries (accessed in August 2022) (34) alongside the Universal Protein Contaminants database (accessed in August 2022) (35), using SEQUEST search (36) and INFERYS deep learning predictions (37). A maximum of two missed cleavages was allowed, with peptide length restricted to 7 to 30 amino acids. Fixed modifications were set to carbamidomethyl (C) and TMT 10-plex (K, peptide N-termini), and variable oxidation (M) modification and deamidation (Q, N) were considered, with the maximum equal modifications per peptide set to 3. Percolator was used to assess the false discovery rate (FDR) with a strict target FDR of 0.01 (38). Standard mass tolerances of 10 ppm and 0.5 Da were allowed for precursors and fragments, respectively.

### DIA Sample Preparation, Mass Spectrometry, and Database Search

A minimum of 8.5 μg of protein from each fraction was reduced by shaking for 1 h with 10 mM of DTT (dithiothreitol) and alkylated by shaking for 2 h in the dark with 25 mM of IAA (iodoacetamide). Samples were precipitated with 6 volumes of acetone at −20 °C overnight.

Precipitated proteins were recovered by centrifugation at 15,000 RCF for 20 min at 10 °C, before removal of the supernatant and resuspension in 25 μl of 100 mM TEAB (Triethylamonium bicarbonate). Each protein fraction was digested with trypsin overnight at 37 °C, with a trypsin to protein ratio of 1:20 (w/w). Desalting was performed using Pierce Peptide Desalting Spin Columns (Thermo) according to the manufacturer’s instructions. For analysis, the fractions were lyophilized and resuspended in 0.1% formic acid.

The sample proteome was measured using a timsTOF HT mass spectrometer (Bruker Daltonics) coupled with a nanoElute 2 UHPLC system (Bruker Daltonics). A total of 750 ng of peptide from each fraction was injected to a PepMap Neo trap column (300 μm × 5 mm, 5 μm particle size, Thermo Scientific) and subsequently separated on a μPAC Neo reverse-phase column (500 mm × 180 μm, 16 μm pillar length, Thermo Scientific) over a 60 min gradient. LC separation was carried out at a flow rate of 300 nl/min using a mobile phase composed of 0.1% formic acid in H_2_O (solvent A) and 0.1% formic acid in acetonitrile (solvent B). The gradient elution progressed from 5% to 17% solvent B over the first 42 min, followed by an increase to 26% over the next 14 min and a final rise to 37% for the last 4 min. Throughout the separation, the column temperature was maintained at 45 °C.

The eluates were ionized using a Captive Spray source equipped with a ZDV Sprayer emitter (20 μm, Bruker Daltonics). The mass spectrometer operated in positive ion mode, covering a m/z range of 100 to 1700. Ion mobility (IM) separation was configured between 0.80 and 1.35 1/K0 [V s/cm^2^], with both the accumulation and ramp times set to 100 ms. Collision energy was dynamically adjusted according to the IM, ranging from 20 eV at 0.60 1/K0 to 59 eV at 1.6 1/K0. The dia-PASEF window settings were optimized for the U-2 OS cell proteome using py diAID version 0.0.19 (39) with a dda-PASEF library as input. A total of 10 dia-PASEF scans were divided into three IM windows, spanning a mass range of 300–1200 Da, with an estimated cycle time of 1.17 s. We did not use an RT standard or randomize our DIA samples. MS1 data were acquired, and the m/z range was not fractionated as single-shot acquisition was used per fraction.

The dia-PASEF data obtained from the mass spectrometer were processed and analyzed using DIA-NN 1.8.2 beta 27 (29) to perform peptide sequence searches against the human SwissProt database with 20,399 entries (accessed in August 2022) (34) alongside the Universal Protein Contaminants database (accessed in August 2022) (35). *In silico* spectral libraries were generated based on the reference databases, incorporating predicted retention times and ion mobility (1/K0) values. A maximum of two missed cleavages was allowed, with peptide length restricted to 7 to 30 amino acids. Up to three dynamic modifications were considered, including methionine oxidation and methionine loss at the protein N-terminus, while carbamidomethylation of cysteine was set as a fixed modification. To minimize missing identifications, the 'match between runs' option was enabled, and we used the QuantUMS (high precision) strategy. Mass tolerance was automatically optimized within the DIA-NN search, and for the DIA-LOP replicates, optimized mass accuracy was 19.7355 ppm, and for the DIA-detergent replicates, it was 11.4744 ppm. An FDR cutoff of 1% was used at the precursor level.

### Data Analysis Workflow

Protein localization analyses were performed using the R Bioconductor packages MSnbase and pRoloc (26, 33, 40).

For DDA experiments, data pre-processing involved the removal of contaminants and quality control filtering based on average reporter ion signal-to-noise, co-isolation interference, and percentage synchronous precursor selection (SPS) mass matches. For the TMT-based experiments, we removed PSMs with an S/N ratio <10 and PSMs with co-isolation interference of 75% or above. Normalisation was performed where each PSM was divided by the sum of all intensities for that PSM (sum normalization). Imputation was performed using k-nearest neighbors (k-NN) before aggregation to the protein level by robust summarization (41).

For DIA experiments, data pre-processing involved the removal of contaminants and ambiguous protein groups, quality control filtering based on a minimum intensity threshold, and global missing value filtration with respect to missingness within a fraction (column) and across the separation profile (row). Fractions with missing data for over 70% of precursors and precursors with fewer than two quantitative values across the remaining fractions were removed from downstream analyses. For imputation, each replicate was treated independently to reduce bias, and each precursor row was imputed based upon its proportion of missingness across all of the fractions within a replicate, with precursors missing greater than two values across all fractions assigned to missing not at random (MNAR) and the rest to missing at random (MAR). Mixed imputation was performed in pRoloc, with the MAR values imputed by k-NN and the MNAR values using the minimum value methodology (min). Precursors were sum normalized before aggregation to the protein level by median summarization.

A base set of manually curated marker proteins were used to define 13 subcellular locations (Supplemental Data 1): cytosol, mitochondrion, nucleus, chromatin, plasma membrane, endoplasmic reticulum, Golgi apparatus, endosome, lysosome, peroxisome, proteasome, ribosome 40S and ribosome 60S. For DIA-LOP, additional curated markers were added to account for the increased number of proteins included in this map. Organelle marker distribution profiles were plotted, PCA and t-SNE dimensionality reduction and clustering were performed and QSep resolution quantitation were performed, as described in: (31, 32, 33). Outliers were systematically removed from the PCA by removing the top and bottom 0.01% of points when ranked in order of principal component 1 and 2 values. Support vector machine (SVM) scores were obtained across all organelles, and these scores were used to tailor the thresholding of each organelle individually. F1 scores were computed as the harmonic mean of the precision (a measure of exactness) and recall (a measure of completeness) on marker proteins. Scaled from 0 to 1, a higher F1 score indicates that the markers are consistently assigned to the correct subcellular localization by the SVM algorithm. SVM classification was carried out as described in (33). Metascape (42) was used to profile Human Protein Atlas localizations (43), Disease Ontology terms (44) and Drugbank entries (45) related to all proteins. Reference protein complexes were curated from the EMBL-EBI Complex Portal (46). Schematic was created in BioRender; https://BioRender.com/swvd1cn.

## Results

### DIA-LOP is a High-Throughput Method for In-depth Subcellular Proteome Mapping

Differential ultracentrifugation (DC) has been used extensively to separate subcellular compartments based upon their sedimentation coefficient to determine the localization of proteins. The previous LOPIT-DC protocol achieved impressive subcellular resolution by utilizing precise cell lysis and DC to generate a high number of fractions to sensitively capture protein profile variation, before performing analysis of the resulting DDA data using the pRoloc Bioconductor package in R (16, 26). Despite this, the previous protocol relies upon stable isobaric tags, such as TMT labels, which increases the cost and lowers throughput due to the requirement to perform offline reversed-phase chromatography to separate peptides and therefore increases the number of samples that need to be run on the mass spectrometer.

Here, we introduce DIA-LOP, a label-free version of LOPIT, in which we apply similar principles to preserve the existing advantages of LOPIT-DC; however, we utilized a data-independent acquisition (DIA) pipeline. DIA-LOP yields high-resolution subcellular maps with increased proteomic coverage by isolating peptides for fragmentation in windows rather than individually, to allow the quantification of all peptides present within the sample. This is achieved while also increasing throughput by using shorter gradients and more streamlined sample preparation without isotope tagging and offline reversed-phase chromatography processing. An optimized mixed imputation strategy is utilized within the pRoloc workflow to deal with the mix of missing at random (MAR) and missing not at random (MNAR) data, which provides the key to interpreting DIA-LOP spatial data generated using a high number of DC organelle fractions, to unlock resolution and depth.

**Fig. 1:**
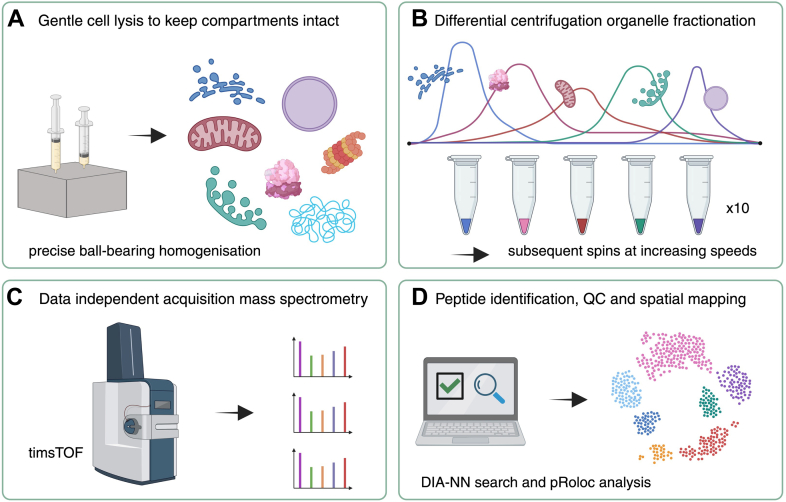
**Schematic overview of the DIA-LOP workflow.***A*, gentle cell lysis via ball bearing homogeniser to keep organelles intact. *B*, organelle separation via differential ultracentrifugation fractionation. *C*, mass spectrometry using data-independent acquisition on a timsTOF instrument. *D*, correlation profiling-based data analysis with peptide identifications, quality control (QC) and spatial mapping using semi-supervised machine learning classification.

DIA-LOP was used to study the well-characterized human osteosarcoma U-2 OS cell line (Fig. 1). Triplicates were obtained using a single 245 mm^2^ dish per replicate to provide sufficient material for both DIA-LOP and LOPIT-DC analysis on the same fractions. After harvesting cells, the DC fractionation workflow was performed (16), using a total of 10 spins at increasing speeds and durations, whilst taking the supernatant each time for the subsequent spin (Supplemental Table S1). This fractionation protocol is simple and easy to perform, and the increased number of fractions compared to other protocols gives the potential for elevated resolution, as the resulting protein profiles will be more granular. Each replicate yielded 11 fractions with at least 40 μg of protein. The initial fraction from the 200 RCF spin was discarded to remove any unlyzed cells, leaving 10 fractions for further analysis. Each fraction was divided into two aliquots to be utilized separately in the DIA-LOP and LOPIT-DC workflows, respectively, thus allowing for a direct comparison.

For DIA-LOP, a minimum of 8.5 μg of protein from each fraction was taken for digestion. TimsTOF PASEF analysis of the DIA-LOP fractions resulted in the identification of 10626, 10493, and 10516 protein groups in the three replicates, respectively. After quality control and imputation of missing values, a total of 8562, 8436, and 8414 proteins remained, with 8242 protein groups common across all three replicates.

For LOPIT-DC, a minimum of 25 μg of protein from each fraction was digested and labelled with a 10-plex TMT kit before pooling and offline reversed-phase chromatography processing. LC-SPS-MS3 analysis of the LOPIT-DC fractions resulted in the identification of 8549, 8319, and 9165 protein groups in the three replicates, respectively. After quality control and imputation of missing values, a total of 6621, 7182, and 6435 proteins remained, with 5777 protein groups common across all three replicates. We observed high reproducibility between replicates regarding protein yield per fraction (Supplemental Fig. S1).

**Fig. 2:**
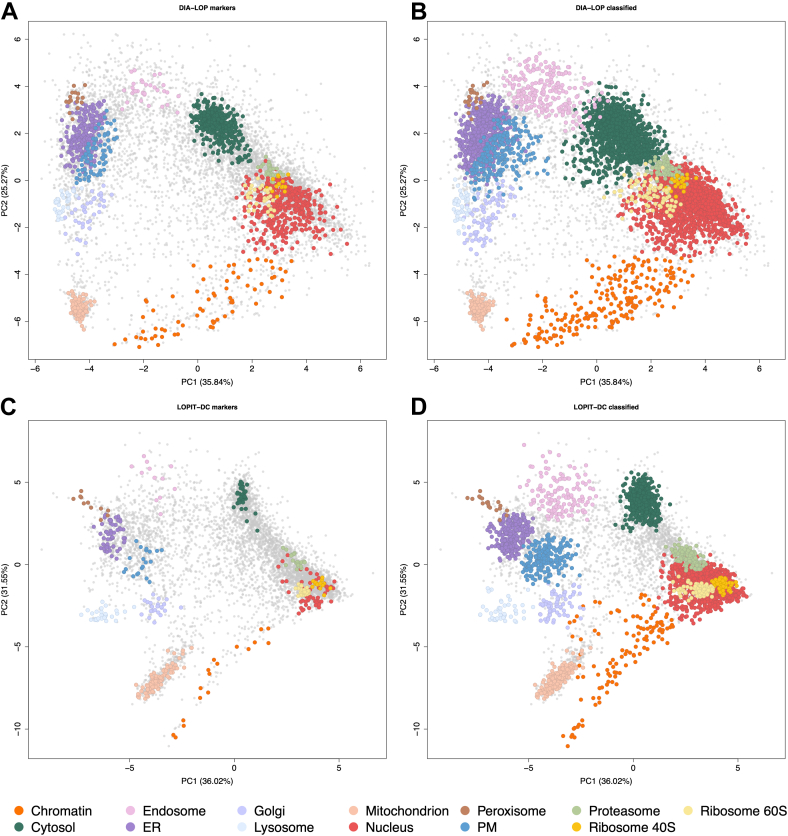
**DIA-LOP resolves all major subcellular niches with a high number of proteins identified.** Principal component analysis (PCA) projections of (*A*) the DIA-LOP dataset with marker proteins annotated, (*B*) the DIA-LOP dataset with unknown proteins classified using support vector machine (SVM), (*C*) the LOPIT-DC dataset with marker proteins annotated, and (*D*) the LOPIT-DC dataset with unknown proteins classified using SVM. Each point represents one protein group. *Grey* points denote proteins that are not marker proteins and do not receive an assigned location after thresholding. PM, plasma membrane; ER, endoplasmic reticulum.

Principal component analysis (PCA) was used to reduce the dimensionality of the data and visualize the variance of protein profiles within a single map, allowing the identification of clusters formed by proteins that belong to the same subcellular compartment (Fig. 2). The DIA-LOP protocol successfully resolved 13 subcellular compartments (Fig. 2*A*), including the nucleus, endoplasmic reticulum (ER), Golgi apparatus, mitochondria, cytosol, plasma membrane (PM), endosome, lysosome, chromatin, proteasome, peroxisome, and both 40S and 60S ribosomes. As expected from using the DC separation gradient, compartments and complexes with similar sedimentation coefficients after cell lysis were clustered more closely on the map, with nuclear and large protein complexes placed together, and secretory membranous organelles forming a separate sector of the map, both resolved considerably from the cytosol and the mitochondrion. The large complexes that clustered closely together within the first two principal components were distinguished in other dimensions, including 40S and 60S ribosomes, and the proteasome (Supplemental Figs. S2 and S3).

When DIA-LOP was compared to LOPIT-DC (Fig. 2, *A* and *C*), both maps showed impressive resolution of the 13 compartments, and the maps displayed a similar overall shape and cluster positioning. This was to be expected since the maps were generated from the same fractions. We found that as DIA-LOP gave rise to many more proteins, we were able to define a greater marker list to use as training data for downstream machine learning.

After observing that the protein profiles of subcellular marker proteins were clustered in PCA space, we concluded that these markers were good candidates to use as labeled training data for supervised machine learning (ML). Both datasets were annotated with a base set of organelle markers (Supplemental Data 1). As the DIA-LOP method detected a far larger number of protein groups, the marker set for DIA-LOP was scaled and supplemented with additional manually curated markers to avoid underlabeling. A support vector machine (SVM) was used to predict the localization of proteins that were unlabeled (Fig. 2, *B* and *D*). This analysis was performed in parallel for DIA-LOP and LOPIT-DC using the 13 aforementioned organelle classes. SVM scores were obtained across all organelles and used to set a class-specific threshold. Overall, DIA-LOP was able to localize 4972 proteins, whereas LOPIT-DC localized 3111 proteins, representing 60% and 54% of their respective total datasets.

Our tailored quality control filters and imputation strategy, combined with our fractionation and MS approach, allowed for the highest number of protein identifications compared to any other DIA subcellular mapping method (Table 2). Compared to other protocols within the literature, we were also able to resolve more subcellular compartments with the use of an increased number of fractions. DIA-LOP can classify a high number of compartments, including separate classifications for the nucleus, cytosol, and chromatin, which was not achieved in label-free DOMs (21).

**Table 2:** A comparison of key DIA-based subcellular proteomics mapping methods, in terms of map size, number of compartments resolved and technical considerations

Method	Number of proteins	Number of fractions	Number of compartments
DIA-LOP	8242	10	13
Detergent (Martinez-Val *et al.*, 2021)	6952	6	12
Label-free DOMs (Schessner *et al.*, 2023)	6571	6	11

DIA-LOP is listed alongside the detergent method (22) and label-free DOMS (21).

### DIA-LOP Achieves High Subcellular Resolution and Increases the Depth of Profiling

**Fig. 3:**
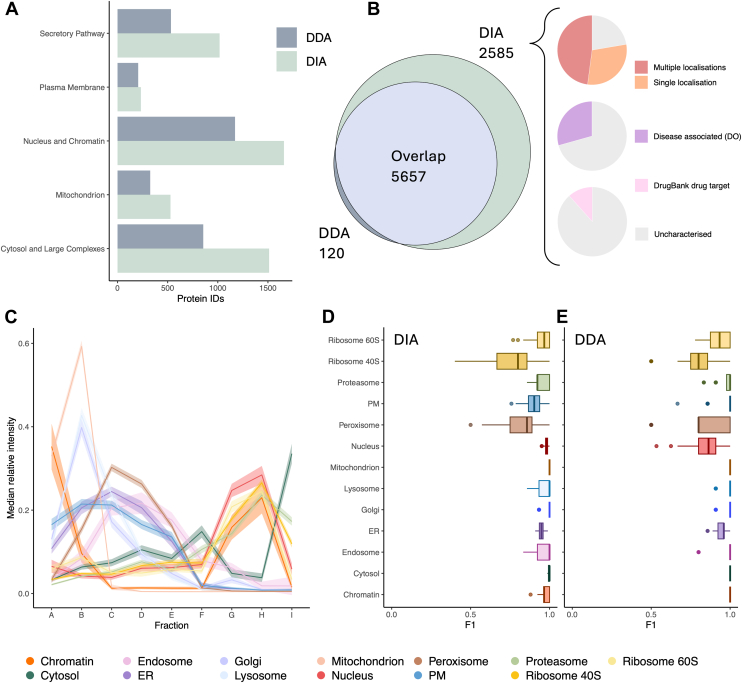
**DIA-LOP performs highly in terms of subcellular resolution and depth.***A*, comparison of the number of protein identifications found in each subcellular region. Subcellular regions are defined as: secretory pathway, plasma membrane, nucleus/chromatin, mitochondrion and cytosol/large complexes. *B*, overlap in protein identifications between DIA and DDA, with distributions of the DIA exclusive proteins shown concerning Human Protein Atlas localizations, Disease Ontology, and Drugbank association. *C*, median marker profiles for organellar markers along all subcellular gradient fractions within a single experiment. Shaded regions denote ± one standard error. *D*, distribution of F1 scores of protein markers from each localization using support vector machine classification for DIA-LOP and (*E*) for LOPIT-DC. F1 scores quantify resolution per organelle. PM, plasma membrane; ER, endoplasmic reticulum.

DIA-LOP identified more proteins in most compartments compared to LOPIT-DC, and similar distributions of the classification of protein groups across each organelle were observed in both DIA-LOP and LOPIT-DC methods (Fig. 3*A*). Most of the proteins identified using LOPIT-DC could also be found using DIA-LOP, with an overlap of 5657 proteins and an extra 2585 mapped using DIA-LOP (Fig. 3*B*). These extra proteins are listed in Supplemental Data 2 and overlaid onto the DIA-LOP map in Supplemental Figure S4. The increased number of protein identifications suggests that the DIA-LOP technique may be able to identify peptides more reliably toward the lower end of the dynamic range (47), since they are no longer filtered out due to the stochastic nature of selective DDA ion fragmentation (Supplemental Figs. S5 and S6). We performed database searches to elucidate the importance of the proteins found only in DIA-LOP (Fig. 3*B*). Comparison with the Human Protein Atlas (HPA) revealed that over half of the additional 2585 proteins have been shown to localize to multiple compartments of the cell, which suggests that some of these proteins could be involved in dynamic cellular processes or display moonlighting properties with different functional roles in different organelles. Over a quarter of these extra proteins are associated with Disease Ontology terms, and likely have roles in pathology. Over 10% of this protein set is also associated with drugs in the DrugBank and could be potentially targeted in future therapies. The top functional enrichment terms reveal that these extra proteins play important roles within protein regulation, including protein localization to an organelle and protein modification by small protein conjugation or removal, as well as essential cellular processes including, carbohydrate derived biosynthetic processes, DNA metabolic processes and regulation of the cell cycle (Supplemental Fig. S7). This highlights the importance of capturing a wider pool of protein groups across all replicates to maximize the depth of the spatial map whilst avoiding the loss of essential information and is facilitated by the increased acquisition consistency and depth of DIA. Expanding the scope of spatial maps will make them more useful in the study of essential cell processes, as well as widening the application of subcellular proteomics within biomedical and drug-discovery settings.

To assess the resolution of the DIA-LOP data, multiple methods were employed. The profiles of marker proteins were plotted to initially deduce the separation between different subcellular compartments (Fig. 3*C*). We observed a high degree of profile separation, matching the PCA visualization. During SVM model training, five-fold cross-validation was employed on the marker proteins, and class-specific F1 scores were obtained (Fig. 3, *D* and *E*). The F1 score is computed as the harmonic mean of the precision (a measure of exactness) and recall (a measure of completeness), scaled from 0 to 1; a higher F1 score indicates that the markers are consistently assigned to the correct subcellular localization by the classifier. The median macro F1 scores were close to 1 across all subcellular classes for both DIA-LOP and LOPIT-DC, indicating that both methods were able to correctly classify the subcellular markers, suggesting a high degree of resolution for all 13 organelles in both datasets. Both methods indicated that the mitochondrion and chromatin were highly separated when compared to the other subcellular classes in the data. In particular, DIA-LOP excelled at resolving cytosolic proteins and achieved high F1 scores for the secretory pathway organelles and the nucleus. The comparable resolution of DIA-LOP to the LOPIT-DC gold standard, coupled with the increased depth, highlights DIA-LOP as a powerful workflow.

### The DIA-LOP Workflow Provides a Solution to Potential Technical Pitfalls

**Fig. 4:**
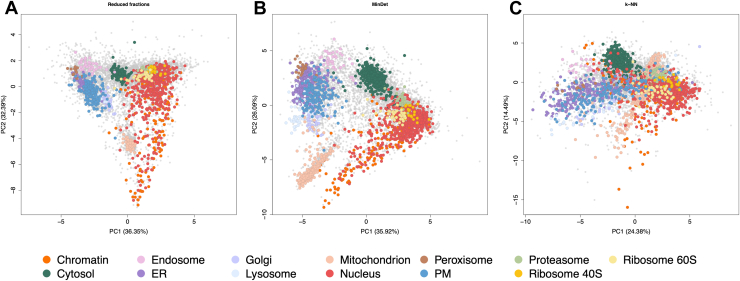
**Highlighting potential problems associated with using suboptimal parameters within a workflow.** PCA projection of (*A*) the DIA-LOP data with fractions removed, to highlight that mapping with a reduced number of fractions gives reduced resolution, (*B*) DIA-LOP data imputed globally with the left censored MinDet method, and with (*C*) the non-parametric k-NN algorithm, to highlight the dangers of erroneous imputation. Each point represents one protein group. Coloured points represent marker proteins as annotated from the literature. *Grey* points denote proteins that are not assigned a location. PM, plasma membrane; ER, endoplasmic reticulum.

It is theoretically known and has been shown from previous studies (48, 49) that increasing the number of fractions in a correlation profiling experiment can increase the resolution of the data. Our robust DC fractionation strategy results in 11 fractions (Supplemental Table S1), which yield sufficient resolution to differentiate the main subcellular compartments of the cell. Here, we ran 10 fractions on the mass spectrometer, discarding the initial fraction of the DC protocol. The first fraction was primarily utilized to remove any unlysed cells, but users can run this first fraction if desired. Figure 4*A* demonstrates the effect on data resolution when a smaller number of fractions is employed. The resolution is lower when fractions are removed because the profiles have far less granularity, meaning that there are a lower number of datapoints to be able to discern the profiles from each other.

One important consideration is that when a higher number of fractions are used, the data may suffer from more missingness. This is because all LOP experiments aim to separate proteins into fractions to generate profiles in which the maximum and minimum intensities across the profile are different for each subcellular compartment. Since optimal separation involves enriching each fraction with a different proportion of proteins from any given subcellular compartment, not all fractions contain proteins from every compartment. Therefore, when the cell lysate is separated into an increased number of fractions, the number of expected missing values increases.

In mass spectrometry-based subcellular spatial proteomics, data missingness may occur for different reasons: (1) missing at random (MAR); wherein missing data arises from the absence of detection of a feature, despite ions being present at detectable concentration and are optimally imputed by ‘hot deck’ methods, which produce unbiased estimates based on similar datapoints across the dataset (2) missing not at random (MNAR); wherein missing data arises from biologically relevant missing values, resulting from the absence or low abundance of ions and are optimally imputed by ‘left-censor’ methods, which assume that the missing data points are below a certain value. We tested the resolution when using baseline methods, such as the *k*-nearest neighbor model (*k-*NN) and a minimum deterministic model (MinDet) (Fig. 4, *B* and *C*). Given the nature of the experiment, both MAR and MNAR points were expected to be present in the dataset; therefore, the regular approach of selecting a global hot-deck or left-censor method was suboptimal, as highlighted on the PCA maps with the marker proteins erroneously overlapping.

To address this issue, we utilized a mixed imputation workflow that couples the *k*-NN algorithm alongside a minimum value strategy (Supplemental Fig. S8). Since technical variation can occur when an individual is performing fractionation, it may be suboptimal to predict whether a point is MNAR or MAR based upon comparison to other replicates, as profiles can be slightly shifted. Therefore, we employed an imputation strategy that involves predicting the likelihood of MNAR or MAR values based upon the proportion of missing values observed across the profile of each protein on a per-replicate basis. This strategy was appropriate because biologically relevant minima are expected where a large proportion of missing values occur across all fractions and are therefore missing not at random. To minimise data imputation, we filtered the data to only retain proteins that were present in at least two fractions of the separation profile on a per-replicate basis. This threshold was selected based on the number of missing values observed across profiles of well-characterized mitochondrial marker proteins. Furthermore, to minimise the instance of false positives, we only considered mapping proteins that were identified in all three replicates. This approach provides the key to interpreting data generated using a high number of organelle fractions, unlocking considerable resolution and depth.

### DIA-LOP Surpasses Detergent-Based Fractionation in Terms of Resolution and Depth

Recently, detergent fractionation has emerged as an alternative approach to gradient centrifugation and DC for correlation profiling. To determine the efficacy of this approach, we performed a detergent-based fractionation workflow (22), for a direct comparison of the resolution within the same experimental pipeline, including similar cell culture, sample preparation, and mass spectrometry analysis. Both DIA and DDA mass spectrometry were performed after detergent fractionation (to produce separate DIA-detergent and DDA-detergent maps), and the data were processed and analyzed as before via the pRoloc pipeline.

**Fig. 5:**
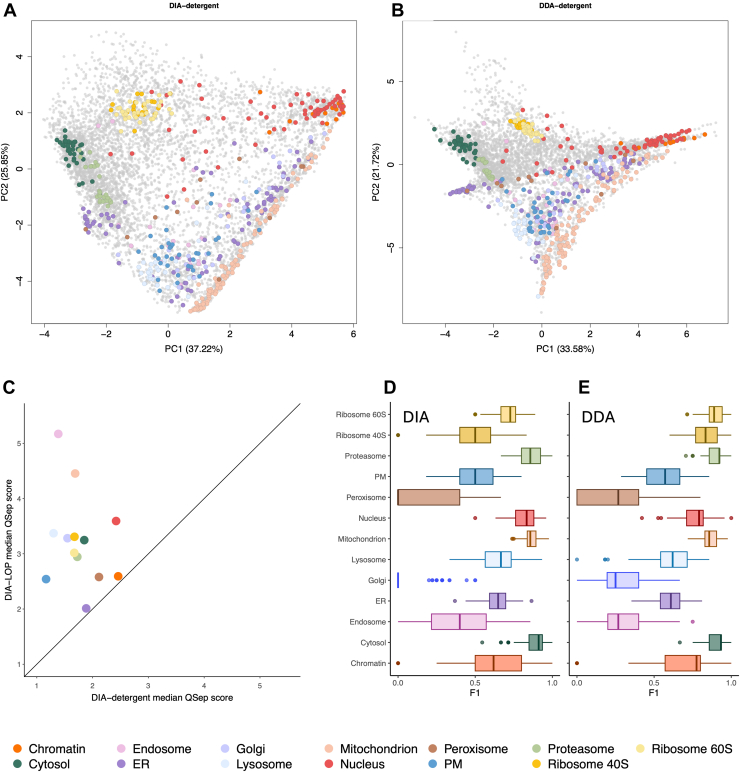
**DIA-LOP outperforms detergent fractionation in terms of resolution and depth.** Principal component analysis (PCA) projection of (*A*) the DIA-detergent dataset and (*B*) the DDA-detergent dataset. Each point represents one protein group. Colored points represent marker proteins as annotated from the literature. *Grey* points denote proteins that are not assigned a location. *C*, median QSep scores plotted to allow comparison of resolution between DIA-LOP and DIA-detergent, with higher scores denoting greater resolution of that organelle. *D*, distribution of F1 scores of protein markers from each localization using support vector machine classification for DIA-detergent and (*E*) for DDA-detergent. F1 scores quantify resolution per organelle. PM, plasma membrane; ER, endoplasmic reticulum.

The processed DIA-detergent replicates contained a total of 6621, 6760, and 6433 proteins, whilst the DDA-detergent replicates contained 6219, 5571, and 6394 proteins. The total number of protein identifications present across all three replicates was 6173 and 5063 proteins for DIA-detergent and DDA-detergent, respectively, which was far fewer than the number identified using DIA-LOP (Fig. 5, *A* and *B*). QSep scores were obtained to enable comparison of the two methods by quantifying the distance between each cluster, alongside the tightness of clustering (Fig. 5*C*). This process relies on the comparison of the average Euclidean distance of the full, n-dimensional protein profiles within and between subcellular marker clusters (32). We observed that the QSep scores for every organelle were higher for DIA-LOP than DIA-detergent, which demonstrates superior resolution of DC compared to detergent fractionation coupled with DIA.

F1 scores were also calculated as a measure of resolution (Fig. 5, *D* and *E*). The cytosol markers formed the most separated cluster, which was also reflected within the class-specific F1 resolution analysis, as this cluster had the highest F1 score. We observed a defined nuclear-associated cluster comprised of both nuclear and chromatin markers; however, these compartments were difficult to distinguish from each other, losing resolution when compared to the DIA-LOP workflow. Similarly, the 40S and 60S ribosomes also formed a cluster in the PCA but were not distinguishable from each other. The main drawback with this fractionation methodology appeared to be the separation of the secretory pathway, namely, the Golgi, ER, endosome, and lysosome, which had limited resolution. This result was to be expected as the membranes of each of these compartments have similar biochemical composition and would likely all interact similarly to any given detergent. Overall, detergent fractionation resulted in lower resolution than DC fractionation when performed within the same experimental pipeline. Of note, the throughput of both methods was similar, as they both required only a single day of experimentation.

### DIA-LOP Provides Insight Into Protein Complexes and Sarcoma-Related Proteins in U-2 OS Cells

**Fig. 6:**
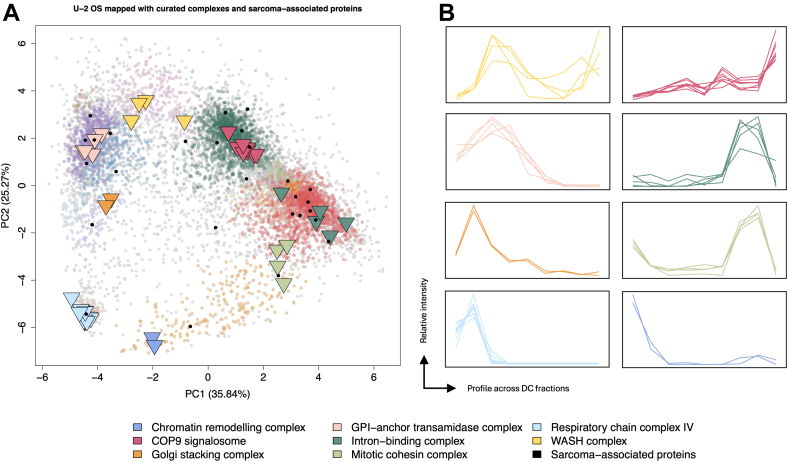
**DIA-LOP can resolve sub-organellar structures, including protein complexes, and can provide disease-related information.***A*, PCA projection of the DIA-LOP dataset, with curated protein complexes overlayed with triangles and sarcoma-associated proteins overlayed with circles. Complexes include the GPI-anchor transamidase complex, respiratory chain complex IV, the COP9 signalosome, ACF chromatin remodelling complex, intron binding complex, GRASP Golgi stacking complex, WASH complex, and the mitotic cohesin complex. *B*, relative intensity linear profiles across the separation gradient of each component present in each protein complex.

Overlaying members of a range of protein complexes onto the U-2 OS DIA-LOP map demonstrated that these complexes cluster well together within the same compartments as expected (Fig. 6*A*), as depicted by the GPI-anchor transamidase complex, respiratory chain complex IV, the COP9 signalosome, ACF chromatin remodeling complex, intron binding complex, GRASP Golgi stacking complex, WASH complex and the mitotic cohesin complex. The profiles across the DC separation gradient were plotted to further demonstrate that the components of these complexes share the same localization (Fig. 6*B*).

When visualized via PCA, we observed that some protein members of the complexes were more separated when compared with their known interactors, which may indicate multi-localization and could represent additional functionality. We noted that the localization of WASHC5, a subunit of the WASH complex, was shifted towards the cytosolic cluster, which could result from its role during the recruitment of the Arp2/3 complex to induce actin polymerization during endosome sorting (50, 51). Furthermore, resolution can be observed for phase-separated organelles; P-body markers formed a distinct region on the PCA map when overlayed, to give a well-defined cluster (Supplemental Fig. S9).

We next demonstrated the power of DIA-LOP to provide clinically relevant information by mapping sarcoma-related proteins within this U-2 OS osteosarcoma cell model (Fig. 6*A*). By identifying the Disease Ontology terms related to every protein in the dataset and searching for the term “sarcoma”, we identified 27 sarcoma associated proteins within the dataset (Supplemental Data 2). Their steady-state subcellular localisations within U-2 OS are highlighted on the DIA-LOP map in Figure 6, and these proteins are mapped using LOPIT-DC in Supplemental Figure S10. The new classifications predicted from the SVM were then compared to other resources, including the Human Protein Atlas (HPA), to allow novel insight, whilst also providing confirmation of existing data (Supplemental Data 2).

70% of our classifications agreed with localizations found in the HPA: including the nuclear localized ERCC1, which plays a role within the nucleotide excision repair pathway by forming a heterodimer with XPF endonuclease and catalyzing the 5′ incision in the process of excising DNA lesions (52); and the cytosol localized small heat shock protein HSPB1, which functions as a molecular chaperone by maintaining denatured proteins (53). Both proteins are thought to play a role in multiple cancers, with the former linked to the efficacy of platinum-based chemotherapies, and the latter thought to promote the evasion of apoptotic pathways (54), which can lead to metastasis (55).

In some cases, our mapping provided more information than the HPA. An example of this was the bromodomain PHD finger transcription factor (BPTF), which is thought to be overexpressed in multiple types of cancer, with poor prognosis (56). Where the HPA entry states nucleoplasm, our SVM mapping indicated that this protein is located specifically in the chromatin.

Six sarcoma-associated proteins were found exclusively in the DIA-LOP dataset and not found in the LOPIT-DC dataset. This included four proteins, which were localized across the cell, in the ER, chromatin, Golgi, and nucleus, as well as two proteins that may be multi-localized, as they were not confidently assigned to a cluster by SVM. The two unassigned proteins were TNF receptor superfamily member 1A (TNFRSF1A), a tumor necrosis factor alpha ligand thought to inhibit inflammation (57), and methylenetetrahydrofolate reductase (MTHFR), which is involved in the remethylation of homocysteine to methionine (58). TNFRSF1A has membrane-bound and soluble forms, which can both interact with tumor necrosis factor alpha (59); however, the HPA does not have confirmed localization data for this protein. In the DIA-LOP dataset, this protein was positioned near the plasma membrane cluster, but slightly shifted toward the other secretory organelles, which could suggest a dynamic role within sarcoma progression.

Interestingly, some of the sarcoma-related protein localization contrasted with data found in the HPA. An example of this is KRAS, a protein linked with 30% of human tumors (60). The HPA suggests that KRAS usually localizes to the cytosol, however, here we associated this protein with the plasma membrane cluster. This could suggest mislocalization of KRAS within the osteosarcoma system, which could mean it is no longer able to perform its role in propagating the kinase cascade, resulting in interrupted cell signaling. Similarly, ASPSCR1, a UBX domain containing tether protein (61), is thought to have a main localization within the nucleus according to the HPA, however, here we localized this protein to the cytosol, which could imply that it is involved in multiple cellular processes. These new subcellular distributions could provide insight into cancerous mechanisms of action within osteosarcoma, for further investigation. This demonstrated one of the ways DIA-LOP can facilitate novel discovery and biomedical impact.

## Discussion

The DIA-LOP method is based upon the LOPIT-DC protocol, a well-established technique for profiling the subcellular spatial proteome and a member of the LOP family of protocols. Despite their capabilities to produce high-resolution maps, all methods within the LOP family currently rely on stable isotope labelling, reversed-phase fractionation, and DDA mass spectrometry, which limits throughput and depth. Here, we expand the capabilities of the LOP family with DIA-LOP, a method that addresses the issues associated with these previous protocols by using DIA-based mass spectrometry. This increases the throughput by removing the need for isotope labeling and reversed-phase offline fractionation, while increasing the coverage by retaining all peptides for fragmentation using wider m/z windows. Importantly, DIA-LOP achieves these improvements whilst retaining comparable resolution to LOPIT-DC.

This study provides a direct comparison between DIA-LOP and LOPIT-DC by using samples prepared in parallel from the same biochemical fractions to give a unique insight into the effects of MS acquisition and instrumentation, coupled with the subsequent diverging bioinformatic decisions required to deal with each data type. We also performed detergent fractionation within the same experimental pipeline to highlight the effect of the chosen subcellular fractionation method on the resolution of the final map. We show that the method of fractionation can drastically affect resolution and depth, as DIA-LOP outperformed the detergent method in several key comparison metrics, including total protein number, F1 scores, and QSep resolution quantitation. Furthermore, in comparison to prominent published DIA-based papers, including label-free DOMs (21) and detergent spatial proteomics (22), DIA-LOP can identify an increased number of proteins and achieve resolution of a higher number of subcellular regions, due to the optimized fractionation and MS workflows. DIA-LOP is therefore an attractive option for spatial mapping of the cell that combines high-resolution differential ultracentrifugation-based fractionation with DIA to boost the number of proteins identified.

The number of subcellular fractions a method can successfully utilize is first determined by the fractionation method itself; for example, there are only so many detergents that can be used in tandem. Secondly, for methods in which the fraction number can be increased, including centrifugation, a limit is naturally imposed by the missing value problem. We highlight the importance of the number of subcellular fractions when creating high-resolution maps and address the resulting challenges associated with missing values across the profile by implementing a mixed imputation strategy. This bioinformatic strategy means that DIA-LOP can unlock the extra resolution afforded by using more fractions than other protocols, including DOMs and the sequential detergent method, and can therefore classify proteins to an increased number of organellar compartments.

Aside from the initial goal of subcellular localization, DIA-LOP can go a step further by demonstrating suborganellar resolution. When the core components of protein complexes are overlaid, clear clustering patterns can be observed. Furthermore, we show that DIA-LOP can produce clinically relevant information. Within the U-2 OS cell model for osteosarcoma, we identify and map the sarcoma-related proteins and find interesting nuances in localization that could provide the foundation for further biomedical research.

Overall, DIA-LOP is a powerful addition to the LOP family of protocols and utilize similar well-established fractionation concepts alongside bioinformatic architecture provided by DIA-NN search software and the pRoloc package in R, while improving the throughput and depth. This approach will facilitate the high-throughput validation of protein localization on a proteome-wide level while also being useful for the generation of new hypotheses.

## Data Availability

The mass spectrometry proteomics data have been deposited to the ProteomeXchange Consortium via the PRIDE (62) partner repository with the dataset identifier PXD063082. Information related to the mass spectrometry data can be found in the Supplemental Data.

## Supplemental Data

This article contains supplemental data.

## Conflict of Interest

The authors declare that they have no conflicts of interest with the contents of this article.
