# Supplementary material for: Localization of Organelle Proteins Using Data-Independent Acquisition (DIA-LOP)
Source: Mol Cell Proteomics. 2025 Aug 7;24(9):101047. doi: 10.1016/j.mcpro.2025.101047 (PMC12455123; doi:10.1016/j.mcpro.2025.101047)
Supplement: Supplemental Material [file mmc4.docx]

**DIA-LOP Supplementary Information: Figures and Tables**

**Supplementary Figures**


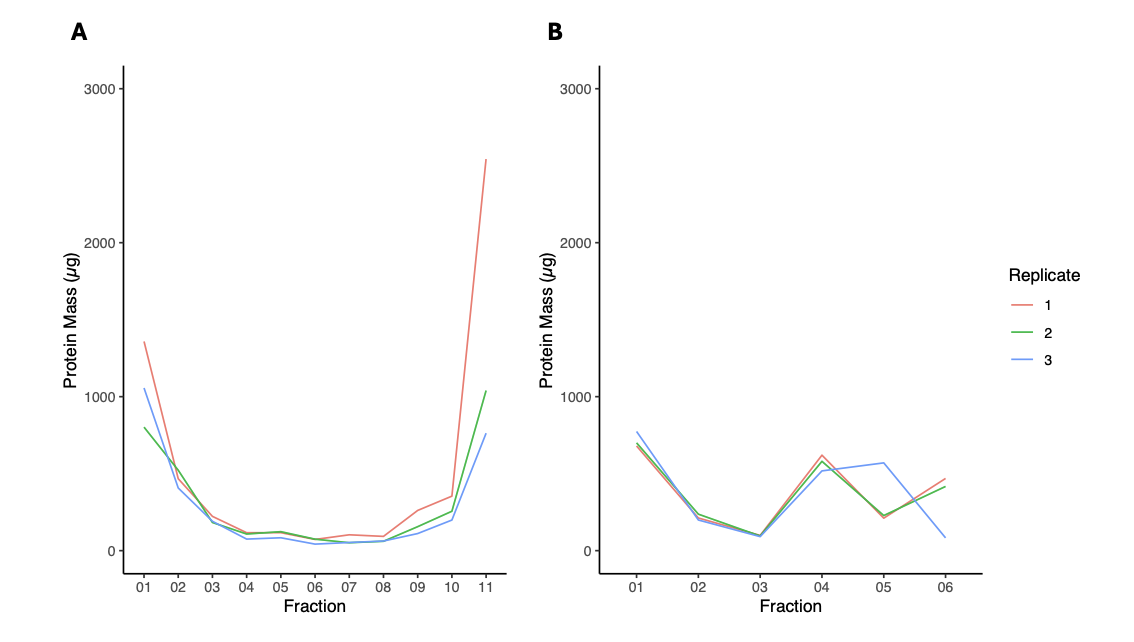


**Supplementary Figure 1 |** Protein yield for all replicates of different fractionation protocols. (A) The distribution of protein mass obtained across all fractions of the differential centrifugation (DC) separation gradient used to generate both the DIA-LOP and LOPIT-DC maps. In the DC data, the final cytosol-enriched supernatant contains the highest protein yield. Larger organelles pellet sooner, and so the first and second pellets contain the next highest protein amounts. Pellet 6 contains the lowest amount of protein. The DC separation gradient shows a high degree of reproducibility. (B) The distribution of protein mass obtained across all fractions of the detergent separation gradient used to generate both the DIA-detergent and DDA-detergent maps.


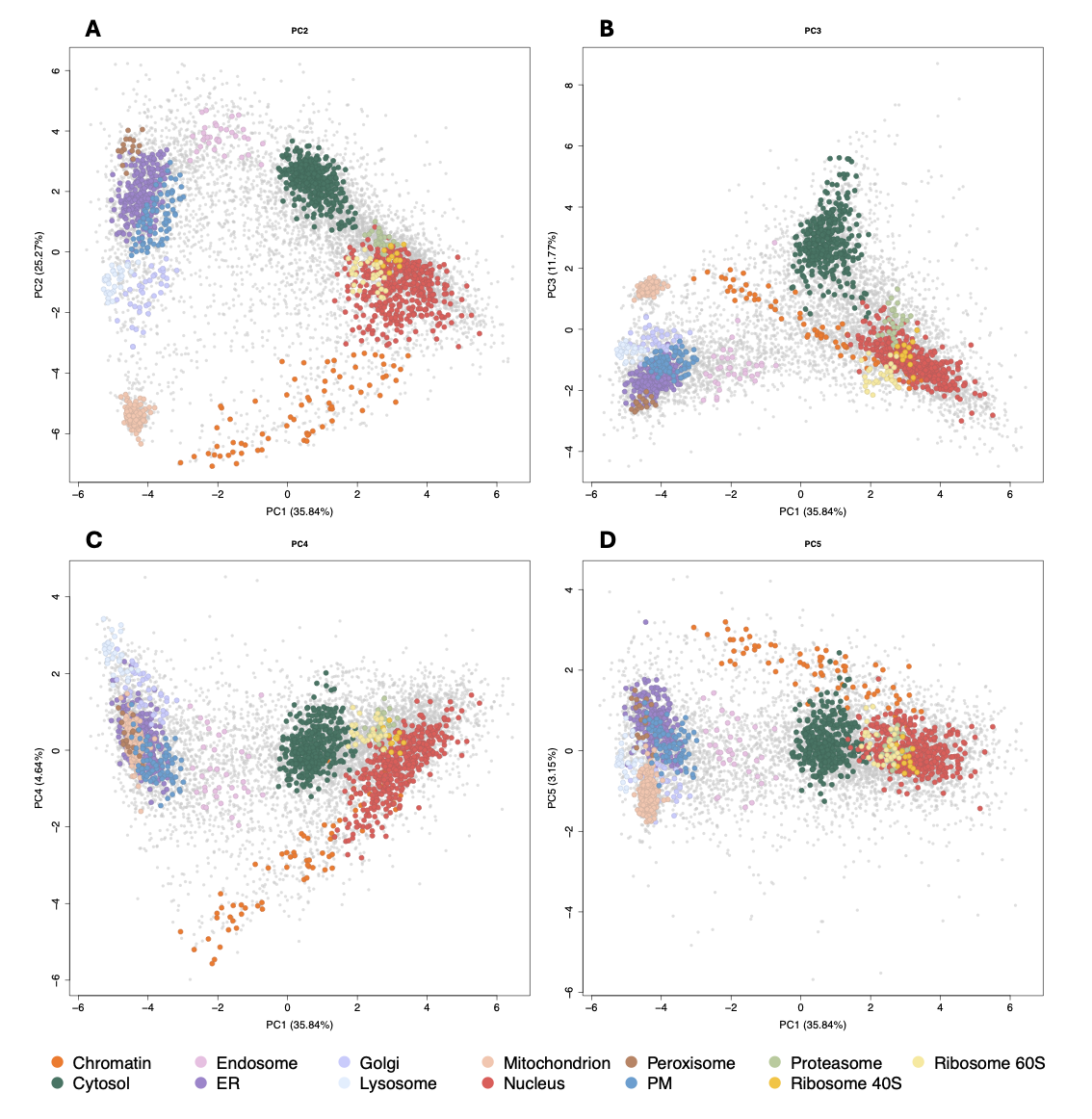


**Supplementary Figure 2 |** Principal component analysis (PCA) projection of the DIA-LOP dataset with the first principal component (PC) plotted against sequential principal components. (A) Standard PCA plot of PC1 vs PC2, (B) PC1 vs PC3, (C) PC1 vs PC4 (D) PC1 vs PC5. Each point represents one protein group. Coloured points represent marker proteins as annotated from the literature. Grey points denote proteins that are not assigned a location. PM = plasma membrane, ER = endoplasmic reticulum.


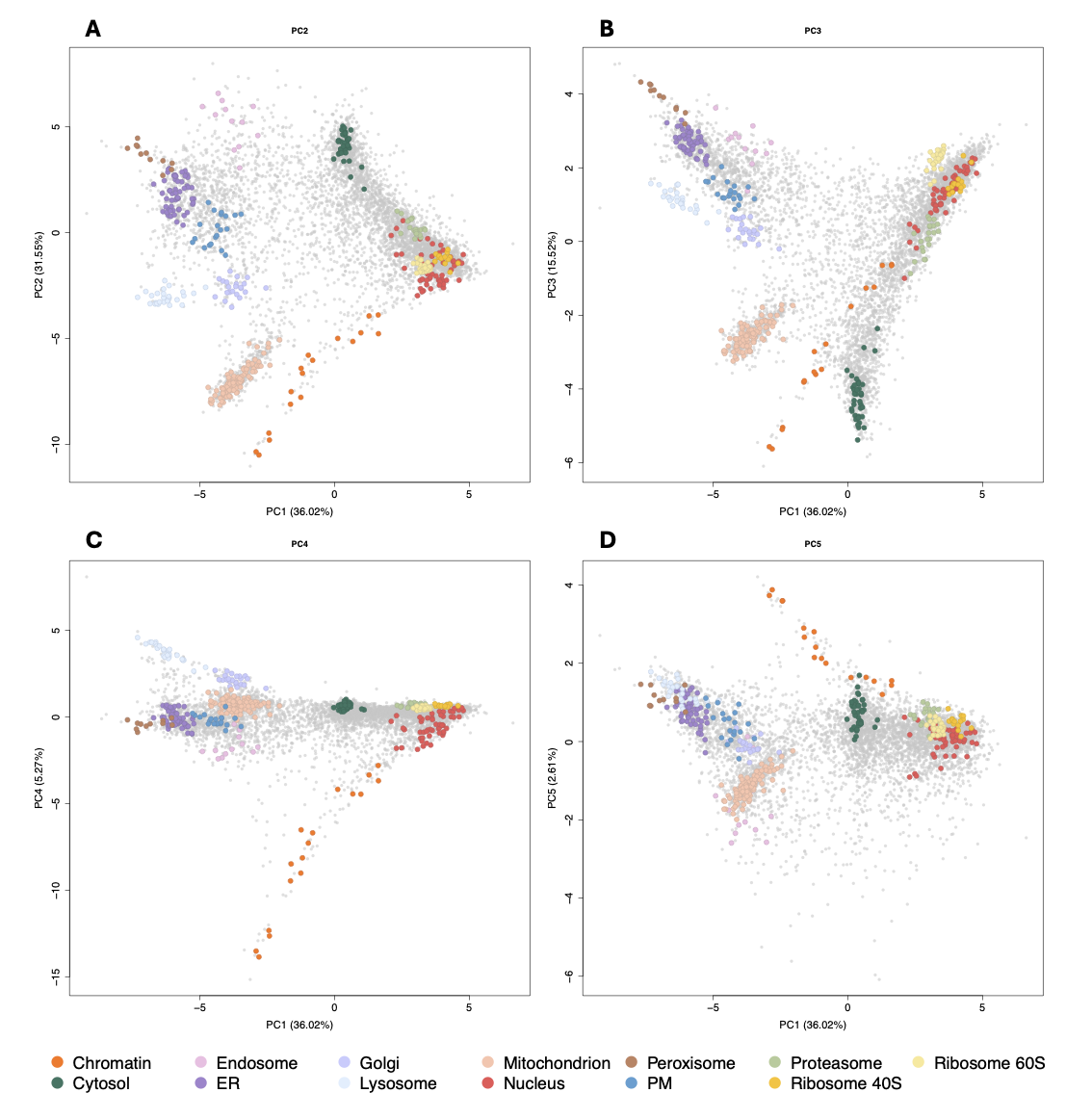


**Supplementary Figure 3 |** Principal component analysis (PCA) projection of the LOPIT-DC dataset with the first principal component (PC) plotted against sequential principal components. (A) Standard PCA plot of PC1 vs PC2, (B) PC1 vs PC3, (C) PC1 vs PC4 (D) PC1 vs PC5. Each point represents one protein group. Coloured points represent marker proteins as annotated from the literature. Grey points denote proteins that are not assigned a location. PM = plasma membrane, ER = endoplasmic reticulum.

**
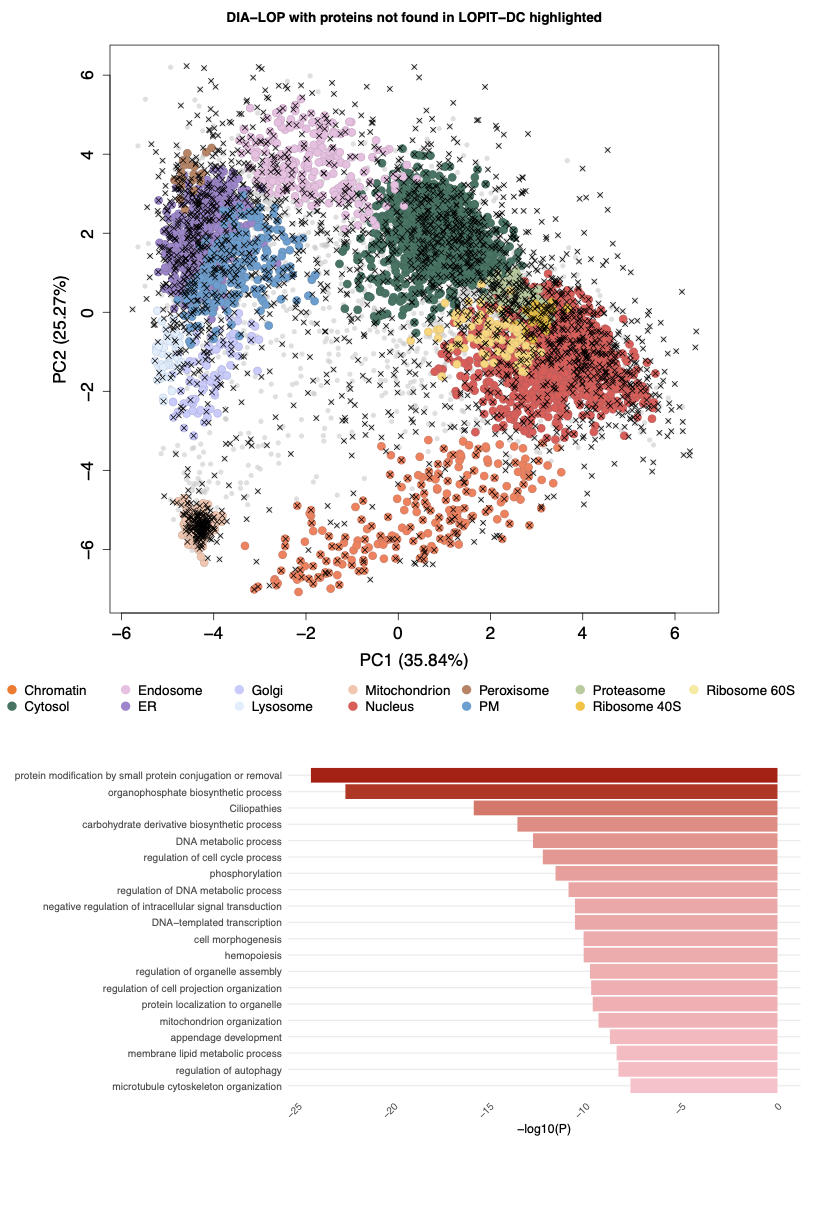
**

**Supplementary Figure 4 |** Principal component analysis (PCA) projection of the DIA-LOP dataset. All proteins that were found in the DIA-LOP map, but not in the LOPIT-DC map are highlighted with an X. Each point represents one protein group. Coloured points represent marker proteins as annotated from the literature. Grey points denote proteins that are not assigned a location. PM = plasma membrane, ER = endoplasmic reticulum.

**
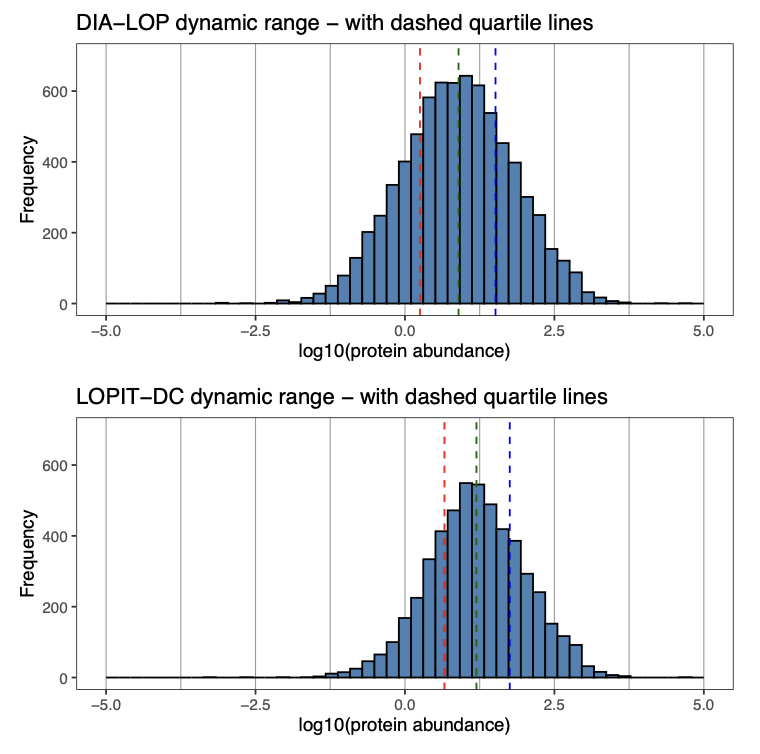
**

**Supplementary Figure 5 |** Distribution of protein abundances detected in DIA-LOP compared to LOPIT-DC, with 0.25, 0.50 and 0.75 quartiles marked in red, green, and blue respectively. This histogram was created using protein identification data from our spatial experiments and combined with abundance data from the PaxDb 5.0 database.

**Supplementary Figure 6 |** Rank-abundance plot to show the dynamic range of DIA-LOP compared to LOPIT-DC. This plot was created using protein identification data from our spatial experiments and combined with abundance data from the PaxDb 5.0 database.

**
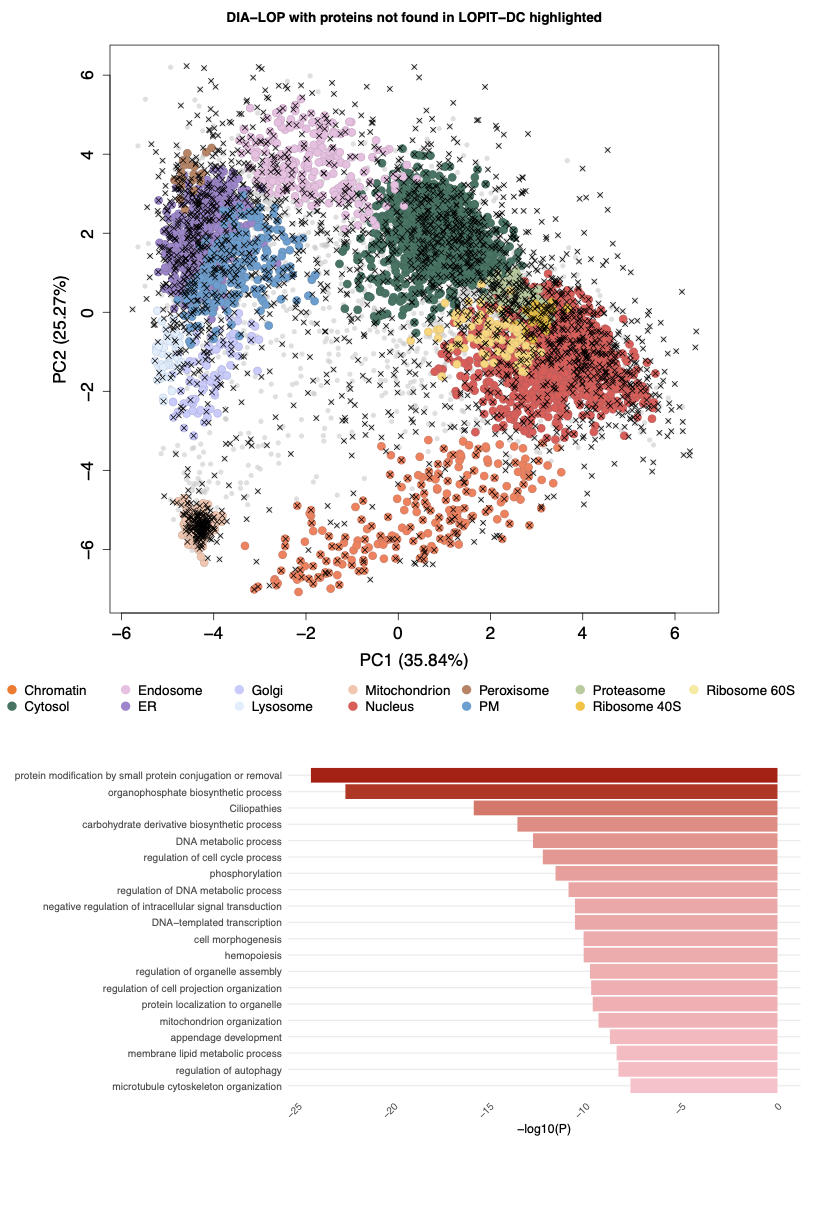
**

**Supplementary Figure 7 |** Gene enrichment analysis of non-redundant terms generated using Metascape, with a color scale to represent statistical significance.


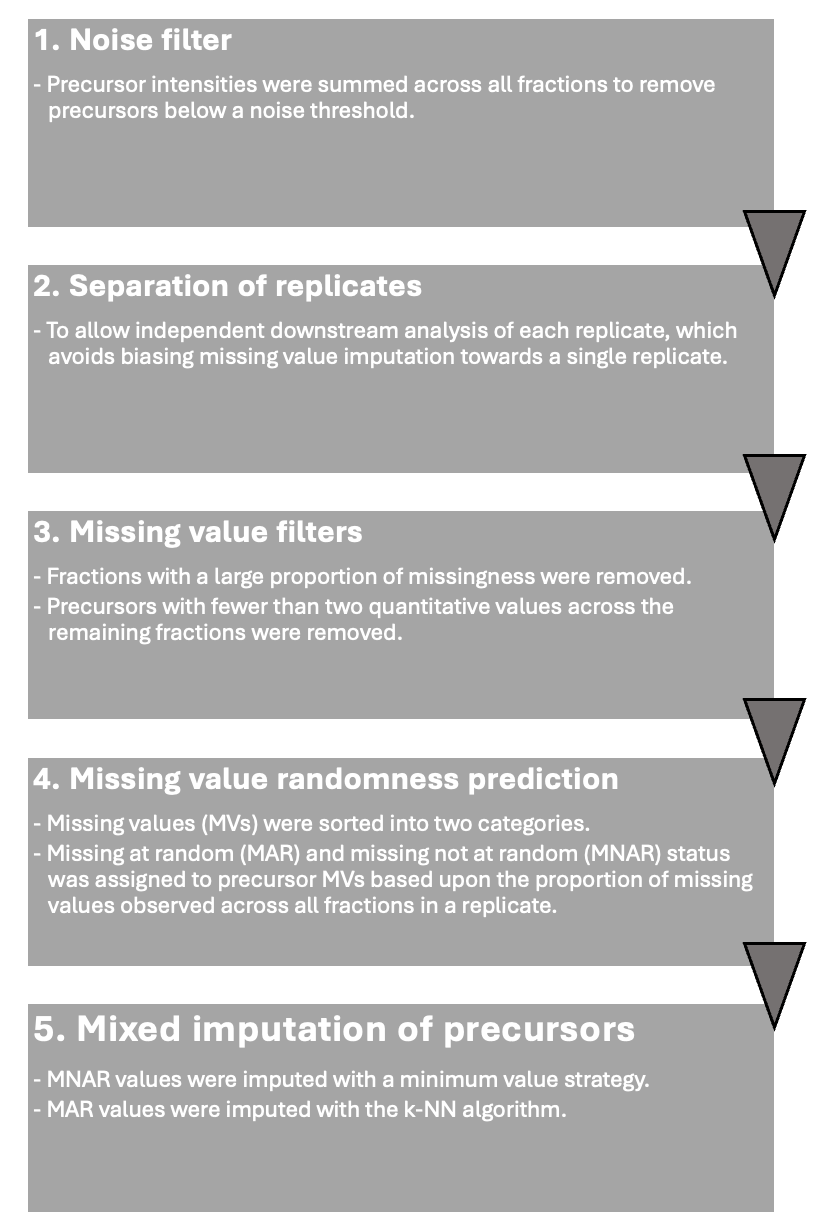


**Supplementary Figure 8 |** Overview of the mixed imputation workflow.

**
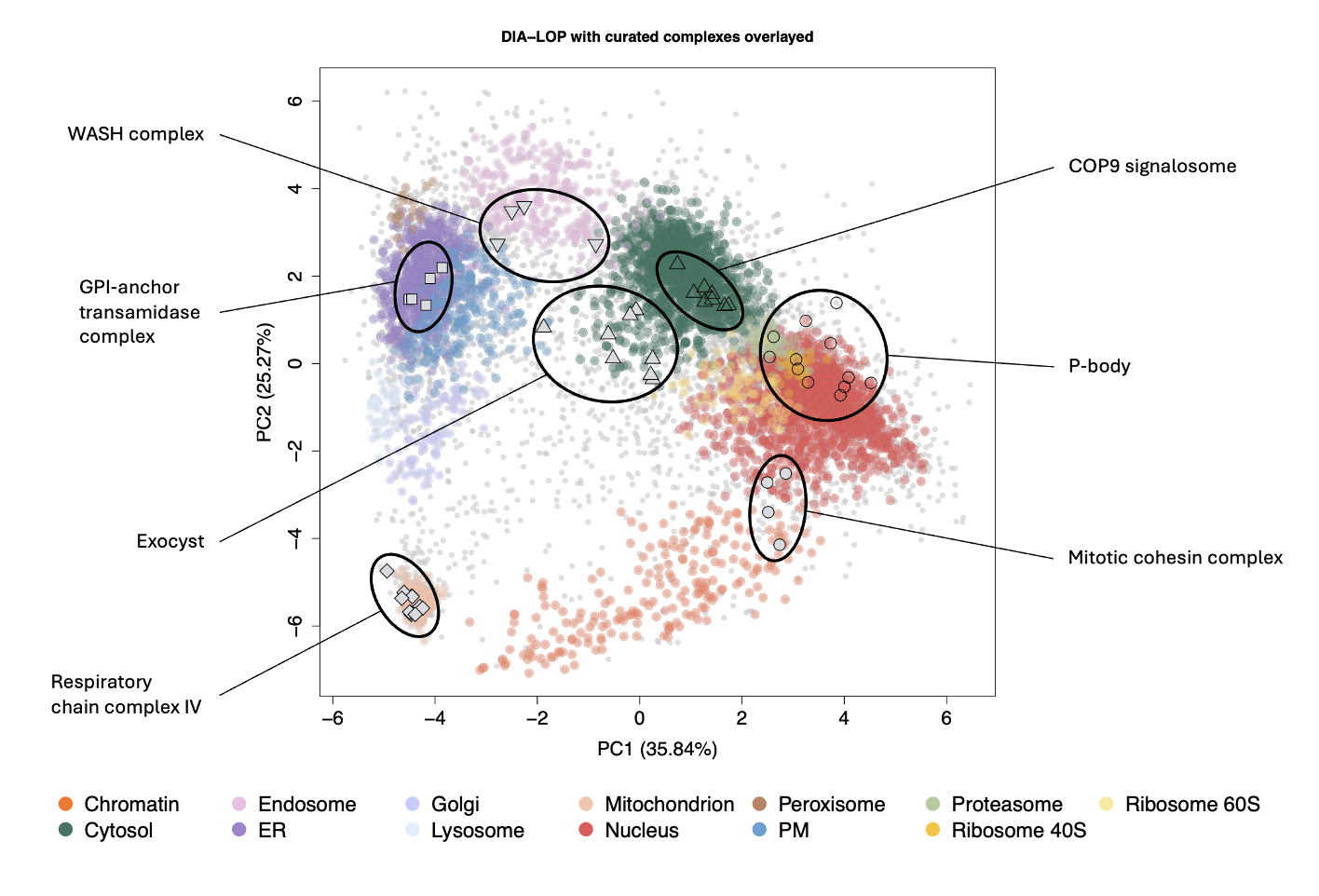
 Supplementary Figure 9 |** Principal component analysis (PCA) projection of the DIA-LOP dataset. Curated complexes are overlayed, alongside P-body markers to demonstrate sub-organellar resolution of phase separated regions. Each point represents one protein group. Coloured points represent marker proteins as annotated from the literature. Grey points denote proteins that are not assigned a location. PM = plasma membrane, ER = endoplasmic reticulum.


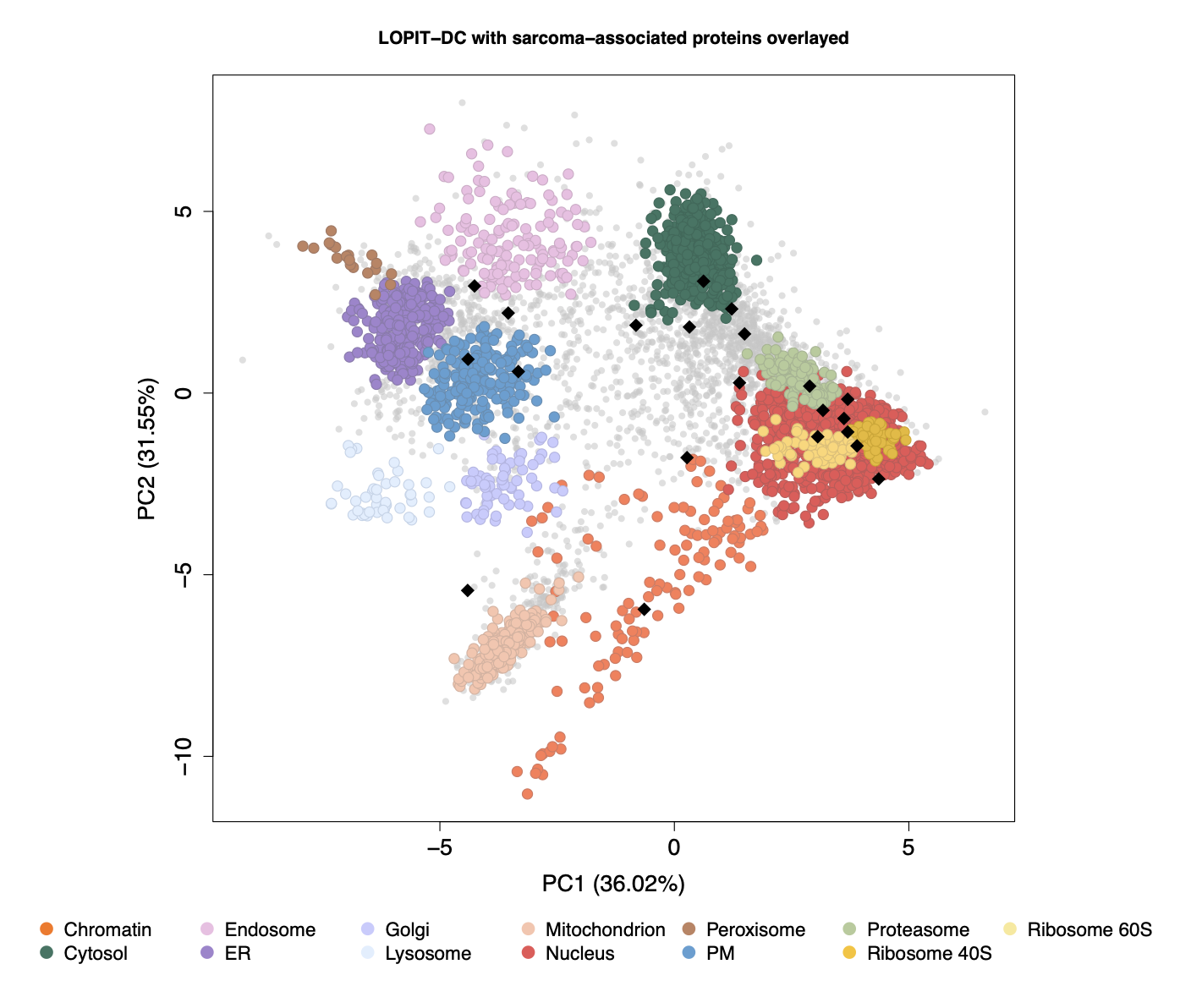


**Supplementary Figure 10 |** Principal component analysis (PCA) projection of the LOPIT-DC dataset. Any proteins that were found to be sarcoma-related in the DIA-LOP dataset, that were also found in the LOPIT-DC dataset have been highlighted here in black. Each point represents one protein group. Coloured points represent marker proteins as annotated from the literature. Grey points denote proteins that are not assigned a location. PM = plasma membrane, ER = endoplasmic reticulum.

**Supplementary Tables**

**Supplementary Table 1 |** Centrifugation parameters for DIA-LOP fractionation.

| **Sample** | **Speed (RCF)** | **Time (min)** |
| --- | --- | --- |
| Initial pellet (MS optional) | 200 | 5 |
| Pellet 1 | 1,000 | 10 |
| Pellet 2 | 3,000 | 10 |
| Pellet 3 | 5,000 | 10 |
| Pellet 4 | 9,000 | 15 |
| Pellet 5 | 12,000 | 15 |
| Pellet 6 | 15,000 | 15 |
| Pellet 7 | 30,000 | 20 |
| Pellet 8 | 79,000 | 43 |
| Pellet 9 | 120,000 | 45 |
| Supernatant (final fraction) | - | - |

**Supplementary Table 2 |** Identification results for the DIA-LOP, LOPIT-DC, detergent-DIA and detergent-DDA datasets.

|  | **DIA-LOP** | **DIA-detergent** | **LOPIT-DC** | **DDA-Detergent** |
| --- | --- | --- | --- | --- |
| Protein groups | 10749 | 8838 | 10065 | 9114 |
| Peptides | 148611 | 97503 | 124990 | 92005 |
| Proteins present in all replicates after filtering | 8242 | 6173 | 5777 | 5063 |
